# The impact of COVID-19 infection, the pandemic and its associated control measures on patients with Pompe disease

**DOI:** 10.1007/s00415-023-11999-2

**Published:** 2023-11-20

**Authors:** Maudy T. M. Theunissen, Renee M. van den Elsen, Tiffany L. House, Brad Crittenden, Pieter A. van Doorn, Ans T. van der Ploeg, Michelle E. Kruijshaar, Nadine A. M. E. van der Beek

**Affiliations:** 1 Department of Neurology, Center for Lysosomal and Metabolic Diseases, Erasmus MC University Medical Center, Rotterdam, The Netherlands; 2 International Pompe Association, Baarn, The Netherlands; 3 Department of Pediatrics, Center for Lysosomal and Metabolic Diseases, Erasmus MC University Medical Center, Sophia Children’s Hospital, Rotterdam, The Netherlands

**Keywords:** Pompe disease, COVID-19, Pandemic, Neuromuscular disorder, Mental health, Patient-reported outcome measure

## Abstract

**Background:**

Patients with Pompe disease, a rare metabolic myopathy, were thought to be at increased risk of severe COVID-19 disease during the pandemic. In addition, the lockdown may have affected their regular treatment.

**Objective:**

To assess the perceived effect of COVID-19 infection and of the pandemic on the treatment, and physical and mental health of patients with Pompe disease.

**Methods:**

Patients with Pompe disease over 16 years of age participated in an international, cross-sectional, online survey (September 20, 2022–November 7, 2022). The questionnaire, available in eight languages, consisted of 89 questions divided into 3 parts: (A) severity of Pompe disease, (B) COVID-19 precautions and infection(s) and (C) effects of the COVID-19 pandemic.

**Results:**

Among 342 respondents, originating from 25 different countries, 47.6% experienced one or more COVID-19 infections. While most recovered within 4 weeks (69.7%) and only eight patients needed to be admitted to the hospital, 42.2% of patients experienced an impact of the infection on their overall condition, respiratory status and/or mobility status. More severely affected patients took more stringent control measures. The pandemic additionally caused interruptions in medical care in many patients (56.0%) and 17.2% of patients experienced interruptions of enzyme replacement therapy. The pandemic also affected many patients’ disease severity (27.7%), mental health (55.4%) and feeling of loneliness (43.4%).

**Conclusion:**

COVID-19 infection(s) and the pandemic affected the treatment, physical health and mental health of patients with Pompe disease, emphasizing the importance of continued patient centered care during a difficult time such as the COVID-19 pandemic.

**Supplementary Information:**

The online version contains supplementary material available at 10.1007/s00415-023-11999-2.

## Introduction

Pompe disease (glycogen storage disease type II, OMIM #232300) is a rare, autosomal recessive metabolic myopathy, caused by pathogenic variants in the GAA gene. These variants lead to a deficiency of the lysosomal enzyme acid α-glucosidase, resulting in glycogen accumulation in the lysosomes—particularly in muscular tissue—, and will ultimately lead to cell destruction [1]. The clinical spectrum of the disorder is broad and continuous. Classic infantile-onset Pompe disease usually presents within the first months of life, with progressive hypotonia, a hypertrophic cardiomyopathy and cardiorespiratory insufficiency [2]. On the other end of the spectrum are the patients with the ‘non-classic’ or ‘late-onset’ form of the disease. This is a more slowly progressive form characterized by symptoms related to axial and limb–girdle weakness of the skeletal and respiratory muscles [3, 4]. The onset can occur anywhere between infancy and late adulthood, and can ultimately lead to wheelchair and/or respiratory support dependency [3, 4]. Since 2006, enzyme replacement therapy (ERT) with recombinant human acid α-glucosidase (alglucosidase alfa; and more recently also avalglucosidase alfa and cipaglucosidase alfa + miglustat) has been registered as a treatment for Pompe disease [5–8]. Despite a considerable variation in individual response, ERT stabilizes or improves most patients’ respiratory and/or motor function. However, a secondary decline is observed in a subset of patients after 3–5 years of treatment [9–14].

COVID-19, an infectious disease caused by SARS-CoV-2, first emerged in December 2019 [15]. The disease, mostly characterized by symptoms such as fever, coughing, fatigue and dyspnea [16–18], has thus far resulted in more than 767 million confirmed cases and nearly 7 million COVID-19-related deaths (last updated July 17, 2023) [19]. Risk factors for a severe course of COVID-19 include male sex, older age, smoking and different comorbidities including obesity, hypertension and cancer [20, 21]. Patients with neuromuscular disorders (NMD), including Pompe disease, were also thought to be prone to a more severe disease course, for example due to cardiac and pulmonary involvement and/or the use of immunomodulatory medication (such as methotrexate or rituximab), as is nowadays often used in many patients with the classic infantile-onset form of Pompe disease to induce immune tolerance to ERT due to the development of IgG antibodies [22, 23]. Several small studies have shown that patients with Pompe disease going through a COVID-19 infection mostly present with mild symptoms and require no hospitalization due to their COVID-19 infection [24, 25]. However, because of the potential vulnerability of this group, they were advised to take additional preventive measures, such as wearing face masks outside of the house, not resuming office work without seeking medical advice and in some cases self-isolation [22, 26].

The COVID-19 pandemic and associated control measures have had a big, though variable, impact on the general populations’ health, including their mental health [27]. It has been shown that in patients with NMDs, including a few patients with Pompe disease, the pandemic resulted in worsening of the quality of life, worsening of their underlying disorder and in some cases in fear and anxiety [28–31]. The COVID-19 pandemic has also been challenging for the disease-specific treatment of NMDs such as ERT, physical therapy and respiratory therapy [28, 29, 32–35].

Research assessing the impact of COVID-19 specifically on patients with Pompe disease is limited. Until now, the mental and physical effects of COVID-19 infection(s), the pandemic and associated control measures remained uncertain. We aimed to learn more about the patients’ experience of these effects through a specifically designed, patient-reported, questionnaire in a large international group of patients with Pompe disease.

## Methods

### Study design and inclusion criteria

This one-time, cross-sectional, questionnaire survey was designed to assess the impact of COVID-19 infection(s), as well as the pandemic and its associated control measures on patients with Pompe disease worldwide. An online questionnaire was created as a collaboration between the Center for Lysosomal and Metabolic Diseases (CLMD) of the Erasmus MC University Medical Center, and the International Pompe Association (IPA), as on request of the IPA. Patients were eligible to participate in this study if they had a diagnosis of Pompe disease, were over 16 years of age and were able to speak one of the eight languages in which the questionnaire was available. All of the outcomes used in this study are patient-reported.

The study design was submitted for approval by the Medical Ethics Committee of the Erasmus MC University Medical Center. It was labeled as not subject to Medical Research Involving Human Subjects Act (WMO) on the June 28, 2022 (MEC-2022-0411). All patients provided online informed consent by checking the consent question in the online survey.

### Data collection and questionnaire design

**Fig. 1:**
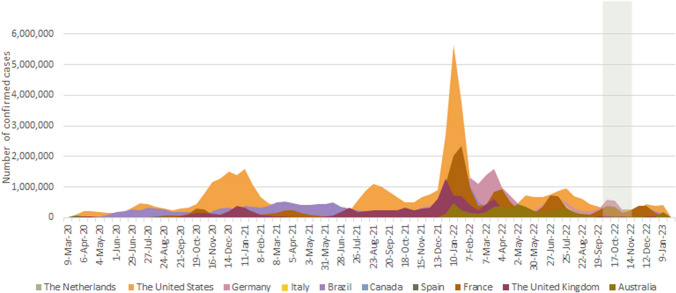
Incidence of COVID-19 infections over time per country since March 2020, when the numbers of cases were rising. The time period in which the survey was sent (September 20, 2022, to November 7, 2022) is highlighted as a vertical column

From September 20, 2022, patients were invited to participate in this study by their national patient organization (Australia, Canada, China, France, Germany, Italy, the Netherlands, New Zealand, Portugal, Spain, UK and USA) or were individually approached by representatives of the IPA. Reminders were sent in several countries, e.g., when the number of responses received was lower than expected. In addition, the survey was advertised on social media, asking the patient to contact designated representatives. The questionnaire was officially closed on November 7, 2022. The time period in which the survey was available is visualized in Fig. 1.

The questionnaire was designed using SurveyMonkey and contained 87 questions. The full questionnaire can be found in Supplementary File 1. It did not ask for any identifiable information except sex, country, month and year of birth, age of onset symptoms and age of diagnosis (for Pompe disease). The content of the questionnaire was based on patient experiences reported by representatives of the IPA and recently published literature regarding COVID-19 and its overall impact on physical and mental health in the general population and in patients with NMDs. Questions were mostly asked in the form of multiple-choice questions and using various formats of a Likert scale, but also yes/no questions and a few open questions were included. The questionnaire was divided into three parts. Part A was designed to collect data regarding the patients’ overall health with respect to Pompe disease. This was based on core elements of the Pompe-specific questionnaire developed for the ongoing annual Pompe survey [36]. Part B was used to assess COVID-19 specific topics, such as vaccination status, precautions taken, the patients’ history of COVID-19 infection(s), the severity of the infection(s) and the effect this had on their mobility and overall and respiratory condition. Lastly, part C was designed to assess the effects of the COVID-19 pandemic on patients’ general health with respect to Pompe disease, their lifestyle, treatment and mental health. The questionnaire was translated into eight different languages (Chinese, Dutch, English, French, German, Italian, Portuguese and Spanish). This was done by the process of forward translation and consensus by two independent IPA associates who have high proficiency in the language of interest.

### Data analysis and patient subgroups

Patients were excluded if they did not answer any COVID-19-related questions. Data cleaning involved fixing and/or removing incorrect and duplicate data. Alleged duplicates were identified by birth year, birth month, sex, country of origin, age of onset symptoms and age of diagnosis. If potential duplicates responded differently on many of the questions in sections A, B and C, or if there were essential differences between them, both responses were retained. Essential differences were defined as differences in certain abilities (e.g., walking, breathing), use of aids, specific years and answers that were on two different ends of a spectrum (e.g., not feeling concerned about the pandemic and feeling very anxious). A consensus meeting (M.T.M.T., R.M.v.d.E. and N.A.M.E.v.d.B.) was held on November 9, 2022, to reach agreement about removing or retaining alleged duplicates. When consensus was met, the response that contained the most answered questions was retained. Both fully and partially completed questionnaires, given that at least one COVID-19-related question was answered, were analyzed in this study. With regard to open questions, consensus meetings (M.T.M.T., R.M.v.d.E. and N.A.M.E.v.d.B.) were held to discuss the correct interpretation of the answers. Some open answers were considered to match an already pre-specified answer and were interpreted accordingly.

Patients were divided into four categories with regard to the severity of Pompe disease. The first category consisted of mildly affected patients, defined as those reporting to use neither breathing aids nor walking aids nor a wheelchair/mobility scooter. Patients who did not use breathing aids, but did use walking aids (or reported not to use walking aids but needing to use one according to their physician) and/or were partially wheelchair-/mobility scooter-dependent, were in the second category and were classified as moderately affected. The third category consisted of severely affected patients, meaning (1) patients using breathing aids solely during the nighttime (or reporting not to use them but needing them according to their physician) or (2) being fully wheelchair-/mobility scooter-dependent. Finally, the last category consisted of very severely affected patients, meaning those reporting (1) using both breathing aids during the nighttime and being fully wheelchair-/mobility scooter-dependent or (2) using breathing aids during the daytime or both day- and nighttime (irrespective of their wheelchair use) or (3) having invasive ventilation (irrespective of their wheelchair use). With regard to age, patients were divided into four age groups: 16–35, 35–50, 50–65 and over 65 years of age.

Descriptive analyses, based on the total number of patients who filled in the question of interest, were performed using IBM Statistical Package for the Social Sciences (SPSS) version 28. The median (interquartile range) was reported for numerical data and the absolute number (%) for categorical data.

## Results

### Patient characteristics

**Fig. 2:**
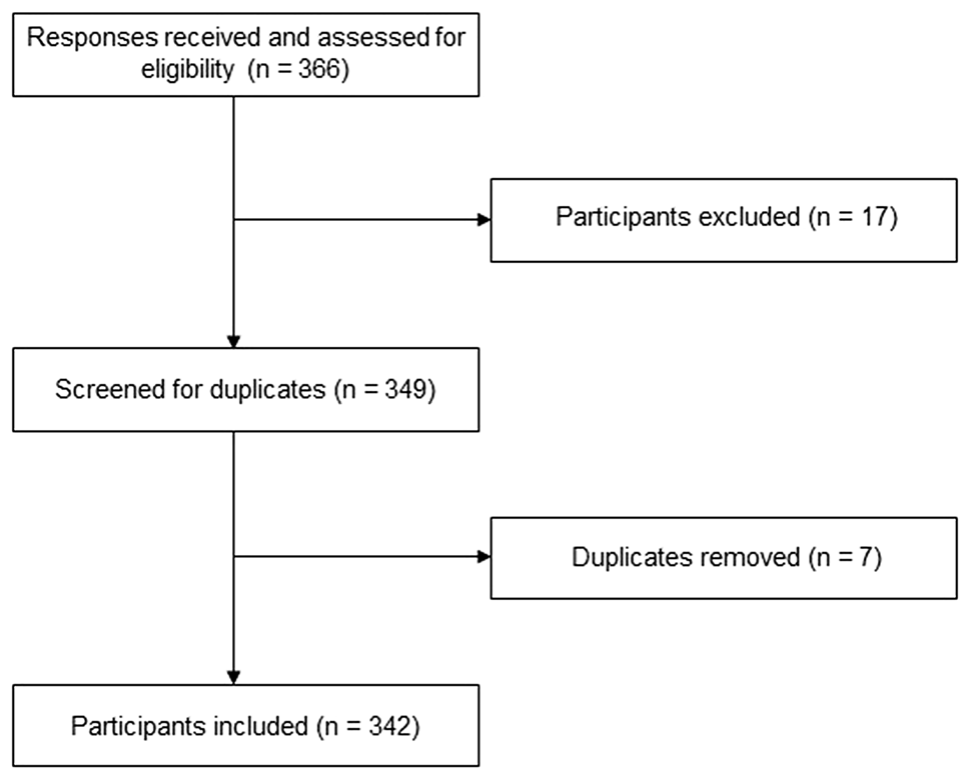
Flowchart of participant inclusion

**Fig. 3:**
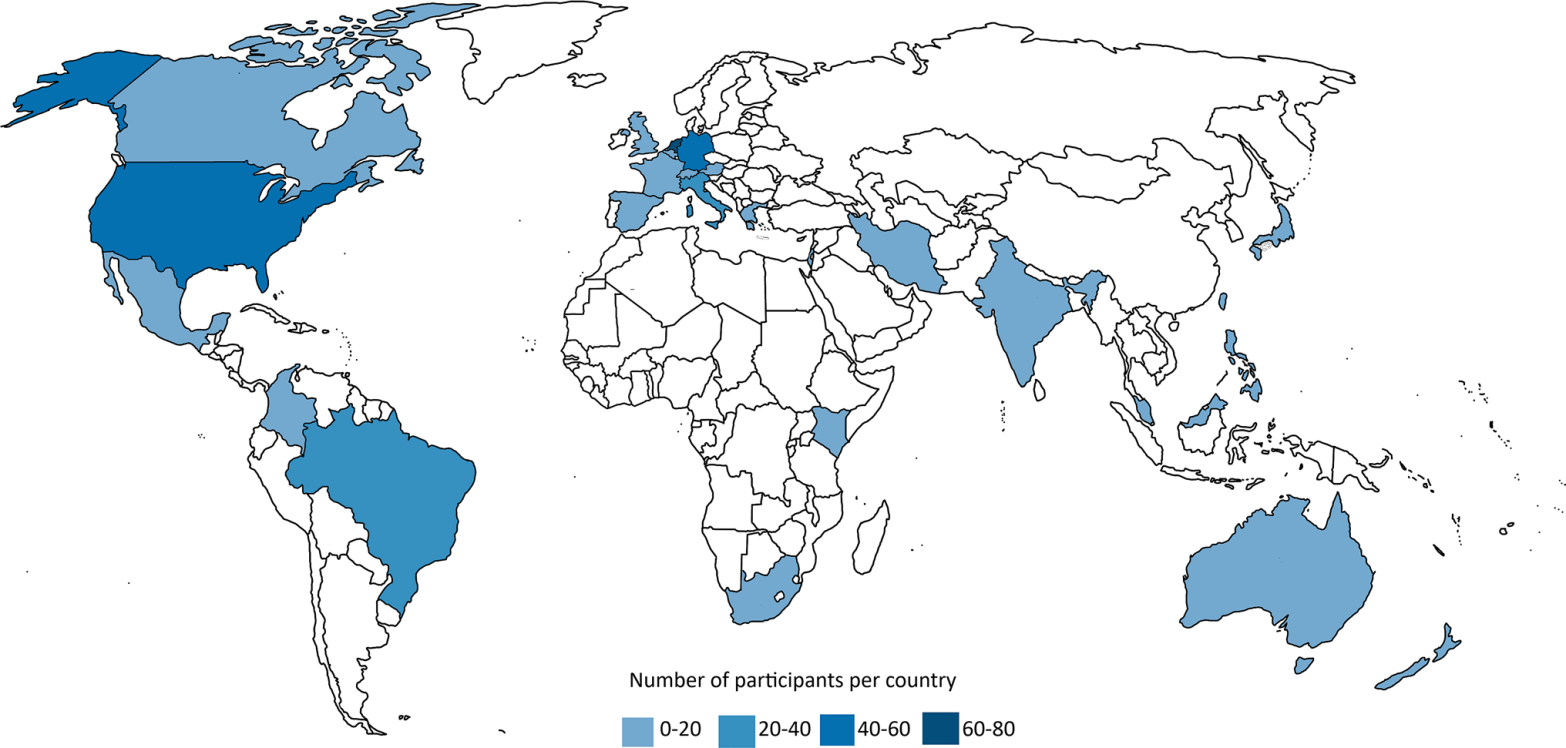
World map of participating countries

A total of 366 responses were assessed for eligibility. Due to the use of multiple platforms to distribute the questionnaire, it was not possible to calculate the average response rate. We excluded 17 patients (no answers to any outcome measures) and removed 7 duplicates (Fig. 2). The final study population consisted of 342 patients. A little over half of patients identified as female (56.7%), the median age was 51 years (IQR 38.0–61.0) and the median disease duration was 22 years (IQR 13.0–31.0) (Table 1). Patients originated from 25 different countries (Fig. 3), the highest number coming from the Netherlands, the USA and Germany. Most patients (*n* = 297) reported being currently treated with ERT. One patient reported to have been treated with gene therapy. Of the 342 patients, 97 were mildly affected by Pompe disease, 46 patients were moderately affected, 121 patients were severely affected and 78 patients were defined to be very severely affected. Two patients reported to need breathing aids according to their physician, but to not have access to them. One of them also needed walking aids according to their physician, but did not have access to them. Both patients originated from Brazil.

**Table 1:** Characteristics of included Pompe patients

Characteristics	Patients with Pompe disease
**Demographics**	
Patients, *n* (% of total)	342 (100.0)
Sex, *n* (%)	
Male	147/342 (43.0)
Female	194/342 (56.7)
Other	1/342 (0.3)
Age, median (IQR), y	51.0 (38.0–61.0)
Age (groups), *n* (%), y	
16–35	68 (19.9)
35–50	83 (24.3)
50–65	131 (38.3)
65 +	60 (17.5)
Country of residence, *n* (%)	
The Netherlands	79/342 (23.1)
The USA	50/342 (14.6)
Germany	42/342 (12.3)
Italy	28/342 (8.2)
Brazil	20/342 (5.8)
Canada	19/342 (5.6)
Spain	18/342 (5.3)
The UK	16/342 (4.7)
France	16/342 (4.7)
Australia	11/342 (3.2)
Other, < 7 patients (Austria, Belgium, Colombia, Greece, India, Iran, Israel, Japan, Kenya, Malaysia, Mexico, New Zealand, Philippines, South Africa, Switzerland, Taiwan)	43/342 (12.5)
**Disease characteristics**	
Age of onset symptoms, median (IQR), y	27.0 (13.0–39.0)
Age of diagnosis, median (IQR), y	36.0 (22.8–46.0)
Disease duration, median (IQR), y	22.0 (13.0–31.0)
Able to walk, *n* (%)	272/342 (79.5)
Without aids	158/272 (58.1)
With aids	114/272 (41.9)
Use of a wheelchair or mobility scooter, *n* (%)	
No	198/342 (57.9)
Sometimes	72/342 (21.1)
Always	72/342 (21.1)
Use of breathing aids (ventilation), *n* (%)	181/342 (52.9)
Noninvasive using a mask/mouth piece	167/181 (92.3)
Invasive ventilation	14/181 (7.7)
Severity of disease (groups), *n* (%)	
Mild	97/342 (28.4)
Moderate	46/342 (13.5)
Severe	121/342 (35.4)
Very severe	78/342 (22.8)
**Treatment**	
Treatment with ERT, *n* (%)	
Never	19/342 (5.6)
In the past	26/342 (7.6)
Currently	297/342 (86.8)

### Frequency, severity and complications of COVID-19 infection(s)

Almost half of the patients (159/334 [47.6%]) reported to have had a confirmed COVID-19 infection (Table 2). Most patients (108/155 [69.7%]) recovered within 4 weeks, while 23.9% (37/155) took longer to recover. Ten patients (10/155 [6.5%]) indicated they were not fully recovered of whom six had a COVID-19 infection within 4 months prior to completing the questionnaire. Of the patients who needed longer than 4 weeks to recover, tiredness and pain were the most common residual complaints.

**Table 2:** Description of COVID-19 infection

COVID-19 infection	Patients with Pompe disease
Confirmed first COVID-19 infection, *n* (%)	159/334 (47.6)
Date first COVID-19 infection, median (min, max)	March 2022 (March 2020–October 2022)
Most common primary symptoms, *n* (%)	
Tiredness	102/157 (65.0)
Nose cold	89/157 (56.7)
Sore throat	87/157 (55.4)
Hospitalized, *n* (%)	8/157 (5.1)
Respiratory support in hospital	2/6 (33.3)
ICU admission	0/6 (0.0)
Rehabilitation necessary in center/rehabilitation home, *n* (%)	2/155 (1.3)
Recovery time, n (%)	
< 4 weeks	108/155 (69.7)
> 4 weeks	37/155 (23.9)
Still not fully recovered	10/155 (6.5)
Most common residual complaints (> 4 weeks recovery time)	
Tiredness	34/44 (77.3)
Muscle pain or other pain complaints	16/44 (36.4)
Coughing or shortage of breath	13/44 (29.5)
Second COVID-19 infection, *n* (%)	19/154 (12.3)
Third COVID-19 infection, *n* (%)	0/19 (0.0)

Eight patients (8/155 [5.1%]) were hospitalized due to their first COVID infection, six of whom were female, seven were severely or very severely affected (by Pompe disease) and five were between 35 and 50 years. All hospitalized patients were vaccinated. No hospitalized patients needed to be admitted to the intensive care unit, although two needed temporarily respiratory support (both female, severely affected and over 50 years of age).

**Fig. 4:**
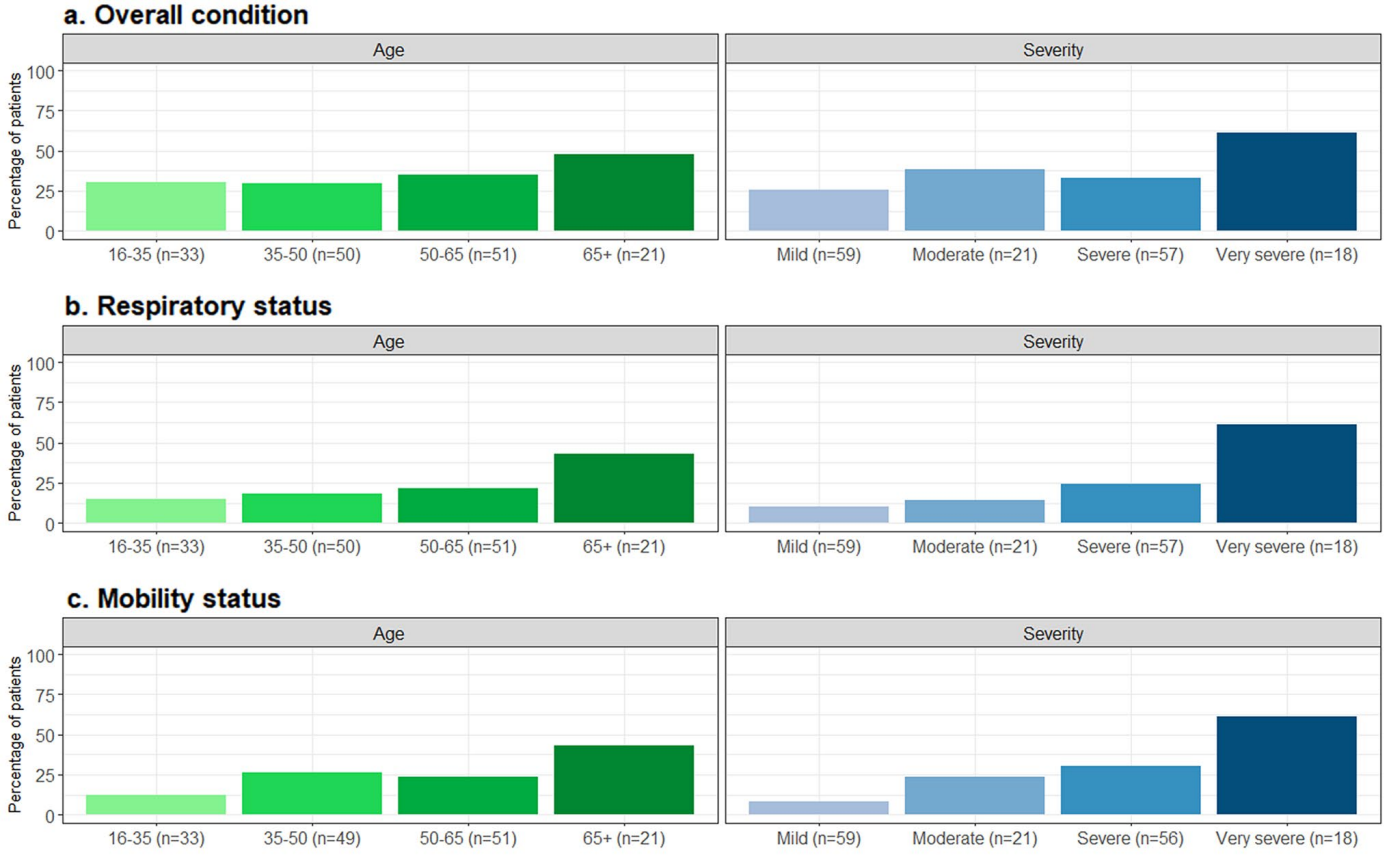
The percentage of patients that indicated that the infection worsened their **a** overall condition, **b** respiratory status and **c** mobility status. Age groups are presented in shades of green, severity groups are presented in shades of blue

Of the patients who had gone through a COVID-19 infection, one in three indicated that the infection worsened their overall condition (53/155 [34.2%]), one in four their mobility status (38/154 [24.6%]) and one in five their respiratory status (34/155 [21.9%]). Over half of patients (89/154 [57.8%]) did not indicate worsening of any of these three domains. Worsening was more commonly reported in patients over 65 years of age and in very severely affected patients (Fig. 4). Similar proportions were observed between sexes (overall condition 34.1% females and 34.3% males; respiratory status 20.0% females and 24.3% males; and mobility status 22.6% females and 27.1% males). Due to their COVID-19 infection, four patients reported needing more hours of ventilator support per day (two of whom reported this was permanent), two patients started using breathing aids (one of whom needed it permanently) and one patient needed the settings of his breathing aids adjusted.

### COVID-19 vaccination and precautions

Most patients received one or more COVID-19 vaccination(s) (326/342 [95.3%]), of whom 309 indicated to have received all recommended doses. This proportion was similar among sexes (95.9% female, 94.6% male). A bigger proportion of mildly affected patients (8.2% mild, 2.2% moderate, 3.3% severe and 3.8% very severe) and patients under 50 years of age (7.9% under 50 and 2.1% above 50) were not vaccinated. Most patients (285/318 [89.6%]) reported also to have received one or more booster doses.

**Fig. 5:**
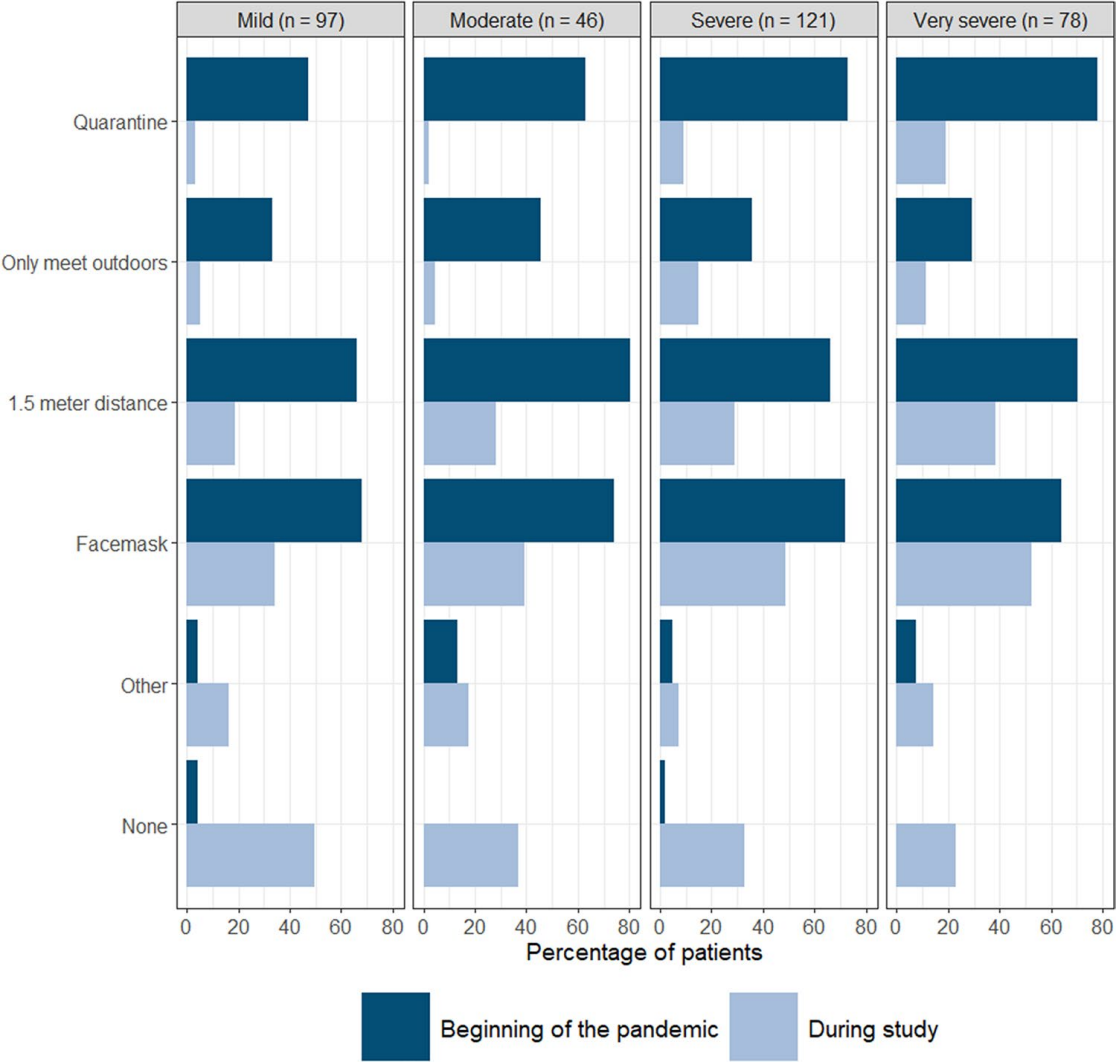
Precautions taken per severity group at the beginning of the pandemic (2020) and during the study (September–November 2022)

At the start of the pandemic, the majority of patients (224/333 [67.3%]) reported they were in quarantine and only met with essential contacts. This precaution was taken by a larger proportion of the severely and very severely affected patients (48.4% mild, 63.0% moderate, 74.6% severe and 82.4% very severe) (Fig. 5). Numerous patients also took more general precautions, e.g., wearing a facemask (237/333 [71.2%]) and keeping 1.5 m distance (236/333 [70.9%]). Among patients who reported to take other precautions, disinfecting hands or objects was most commonly reported. Only six patients (1.8%) reported to take no precautions at the beginning of the pandemic.

When filling in the questionnaire (September–November 2022), a minority of patients (30/333 [9.0%]) still reported to be in quarantine and to only meet with essential contacts. As before, this consisted of a bigger proportion of very severely affected patients compared to other severity groups (3.2% mild, 2.2% moderate, 9.3% severe and 20.3% very severe). Compared to the start of the pandemic, less patients took more general precautions, with wearing a mask when out in public or around those not in their household being the most applied (151/333 [45.3%]). Among patients who reported to take other precautions, disinfecting hands or other objects and avoiding crowds and large groups of people was most commonly reported. In contrast to the start of the pandemic, more patients (123/333 [36.9%]) took no precautions, especially among mildly affected patients (50.1% mild, 37.0% moderate, 33.9% severe and 24.3% very severe) (Fig. 5).

### Impact of COVID-19 pandemic on treatment, physical health and mental health

*Treatment* ERT infusions were interrupted in approx. 1 in 6 patients (56/325 [17.2%]) (range 1–24 infusions, median 3.0 (IQR 2.0–5.3)). In France, this proportion was particularly high (7/14 [50.0%]). A delay in the start of ERT was reported by a few patients (8/325 [2.5%]). Twenty-eight out of 64 patients (43.8%) perceived this interruption/delay of ERT to have negatively affected their condition, mostly negatively affecting their mobility or level of fatigue. This concerned a larger proportion of female patients (54.5% females and 32.3% males) and patients between 35 and 50 years of age (45.5% 16–35, 61.5% 35–50, 22.2% 50–65 and 33.3% 65 +), while a smaller proportion of mildly affected patients reported a negative effect on their condition (35.3% mild, 50.0% moderate, 48.0% severe and 43.8% very severe).

**Fig. 6:**
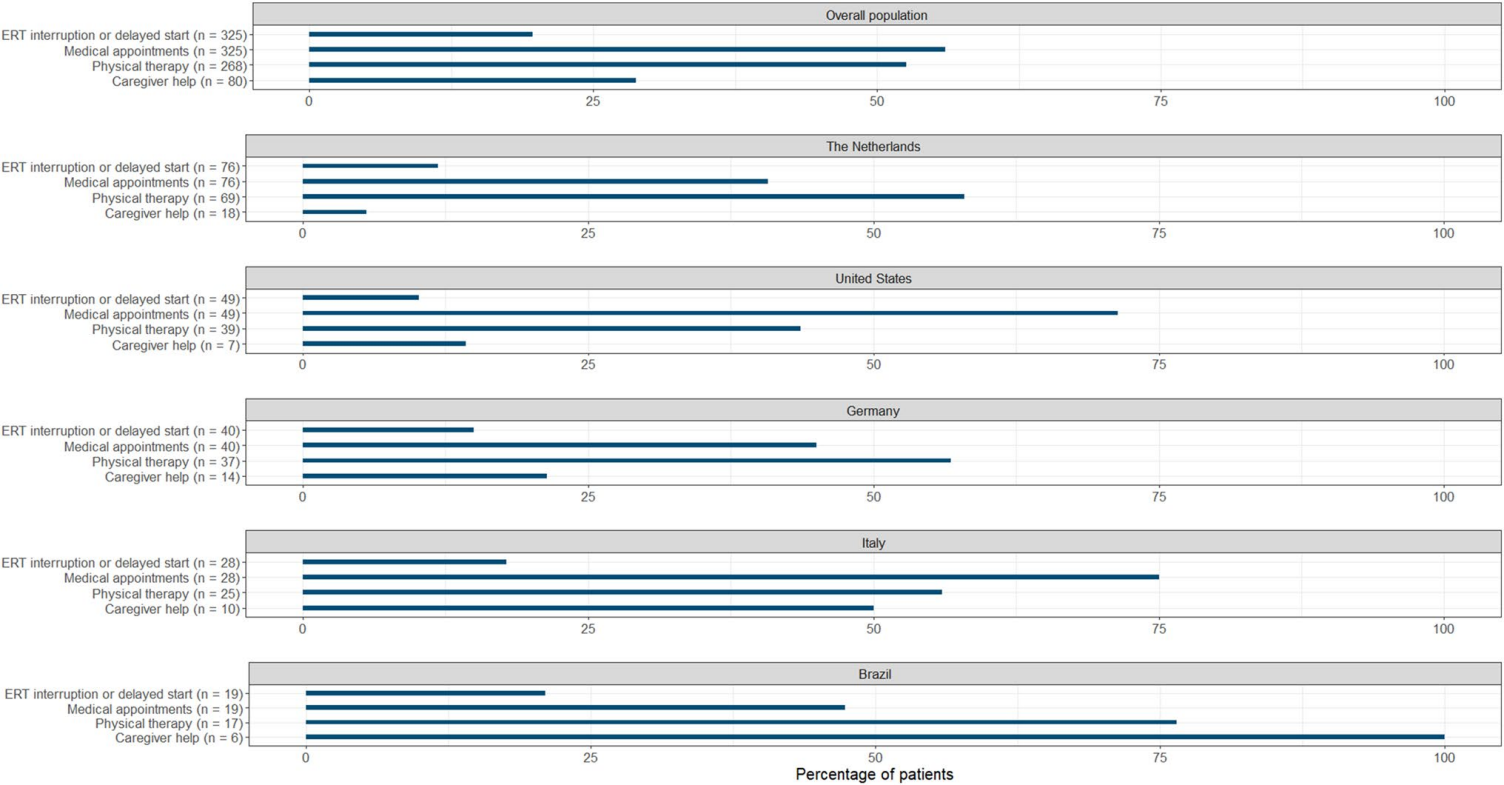
The proportion of patients who indicated a change in their treatment with ERT, medical appointments, physicial therapy and/or caregiver help as a result of the pandemic in the overall population and in the five countries with the most participants

A change in medical appointments due to the pandemic, including less appointments and online consultations, was reported by 56% of patients (182/325). A larger proportion of severely and very severely affected patients reported a change in medical appointments (46.7% mild, 50.0% moderate, 61.7% severe and 62.5% very severe), as well as a bigger proportion of patients from the USA (35/49 [71.4%]) and Italy (21/28 [75.0%]) (Fig. 6). Physical therapy was reported to have been paused once or more than once as a consequence of the pandemic by 52.6% of patients (141/268). The duration of this cessation ranged from 2 weeks to 24 months in total (median 3 months (IQR 1.8–11.0)). Multiple patients reported to receive caregiver help (80/324 [24.7%]), of whom 23 patients experienced reduced visits as a result of the pandemic (28.7%). This change was reported by all 6 patients from Brazil with caregiver help (Fig. 6). The most reported consequence of reduced visits was increased loneliness and/or social isolation.

**Fig. 7:**
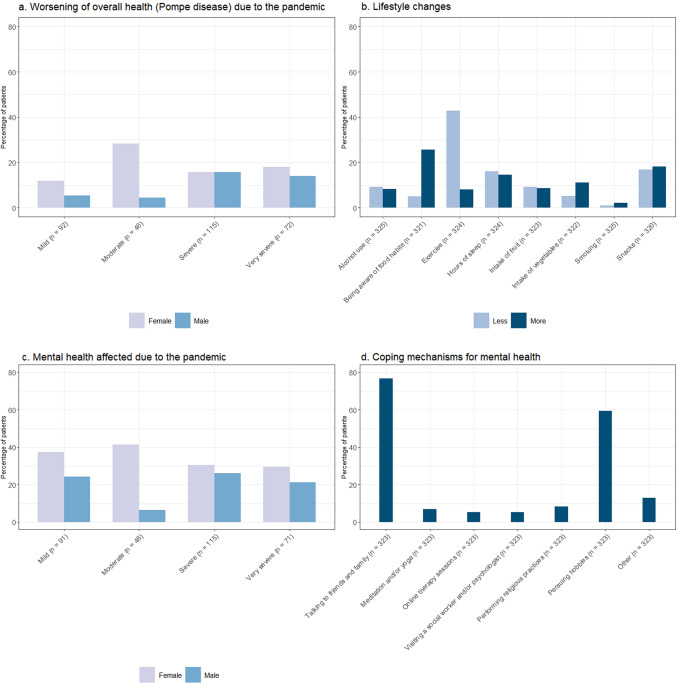

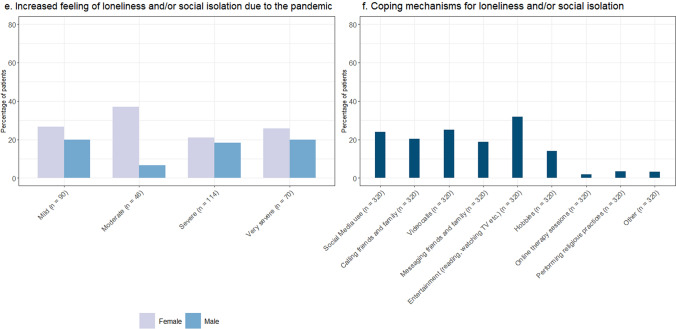
The percentage of patients (with sex ratio) per severity group that indicated that their overall health with respect to Pompe disease (**a**), mental health (**c**) and feeling of loneliness (**e**) was affected due to the pandemic. Lifestyle changes as a result of the pandemic (**b**) and coping mechanisms for their mental health (**d**) and loneliness (**f**) during the pandemic are presented as well

*Overall health with respect to Pompe disease* Compared to the start of the pandemic, 43.1% of patients (140/325) reported that their overall health with respect to Pompe disease had worsened, of whom 90 (64.3%, 27.7% of total) believed this was due to the pandemic and/or lockdown (Fig. 7a). A smaller proportion of milder affected patients (17.8% mild, 16.7% moderate, 40.0% severe and 25.6% very severe) and a larger proportion of woman (61.1% females and 38.9% males) indicated worsening of their disease due to the pandemic. Of the patients indicating worsening of their disease because of the pandemic, 42.2% experienced a COVID-19 infection. The most commonly reported lifestyle changes as a result of the pandemic were: less exercise (139/324 [42.9%]), being more aware of food habits (82/321 [25.5%]), both more and less snacking (58/320 [18.1%] and 54/320 [16.9%]) and a decrease in hours of sleep (52/324 [16.0%]) (Fig. 7b) (see Supplementary File 1 for all possible answer options).

*Mental health* Many patients reported to be mentally affected by the pandemic (179/323 [55.4%]), e.g., experiencing possible symptoms of anxiety or depression or experiencing problems with concentrating or sleeping. A bigger proportion of female patients (60.9% females and 48.6% males) and mildly affected patients (61.5% mild, 47.8% moderate, 56.5% severe and 50.7% very severe) reported that their mental health was affected by the pandemic (Fig. 7c). Feeling nervous, restless or tense was the most common complaint (113/323 [35.0%]) and talking to friends and/or family was the most common reported coping mechanism (248/323 [76.8%]) (Fig. 7d) (see Supplementary File 1 for all possible answer options).

Moreover, a considerable number of patients were reported to have experienced an increased feeling of loneliness and/or social isolation as a result of the pandemic and/or lockdown (139/320 [43.4%]). The proportion of patients experiencing loneliness and/or social isolation was bigger in females (46.4% females and 39.7% males) (Fig. 7e) and smallest in patients over 65 years of age (44.4% 16–35, 52.0% 35–50, 43.1% 50–65 and 32.2% 65 +). Most patients (98/139 [70.5%]) experiencing loneliness and/or social isolation were still taking one or more precautions when filling in the survey. The most often reported coping mechanism was an increase in time spent on entertainment (e.g., reading, watching television, etc.) (102/320 [31.9%]) (Fig. 7f) (see Supplementary File 1 for all possible answer options). In total, 2 in 3 patients (183/278 [65.8%]) indicated to be affected mentally and/or experiencing an increased feeling of loneliness and/or social isolation as a result of the pandemic.

## Discussion

This is the first study to assess how patients over 16 years of age experienced the mental and physical effects of COVID-19 infection(s), the pandemic and its associated control measures in a large international group of patients with Pompe disease. With patient-reported data from nearly 350 patients residing in 25 different countries, this gives a unique insight into the perspectives of patients with a rare disorder. The main results show that at the time of the study, about half of patients had experienced one or more COVID-19 infections. While the majority of infected patients did not require hospitalization and most had a short recovery duration (< 4 weeks), many patients (42.2%) experienced an impact of the infection on their overall condition, respiratory status and/or mobility status. The control measures taken during the pandemic additionally caused interruptions in medical care, physiotherapy and caregiver help in many patients (56.0%, 52.6% and 28.7%, respectively) and approx. 1 in 6 patients experienced interruptions of enzyme replacement therapy. The pandemic also affected many patients’ disease severity (27.7% of all patients indicated worsening due to the pandemic), mental health and/or feeling of loneliness (65.8%). This study shows that the impact of COVID-19 is big though variable and highlights the challenges patients face, which emphasizes the importance of patient centered care.

This cross-sectional survey showed that most patients with a COVID-19 infection recovered within 4 weeks, which is in line with the literature reporting a mild disease course of COVID-19 in patients with Pompe disease [24]. This is also substantiated by our finding that only eight patients reported to have been hospitalized due to their first COVID-19 infection, of whom only two needed respiratory support and none required ICU admission. However, 42.2% of patients in our study with a COVID-19 infection experienced deterioration of their overall condition, respiratory status and/or mobility status. This is in accordance with, or higher than the numbers in, the literature regarding the consequences of COVID-19 infections in patients with other NMDs, which showed that COVID-19 infections caused a worsening of their underlying NMDs in 31.0% of patients [37].

The high frequency of control measures reported in the initial stage of the pandemic, shows that patients were very cautious during the earlier stages of the pandemic, as was advised due to the potential vulnerability of this group [22]. This is also supported by the median date of first infection (March 2022) and the high number of patients in this study reporting to have received vaccination(s). In this study most patients (90.4%) received all recommended vaccination doses, which is higher than the global population average (65.5%) [19]. Also the percentage of patients that received a booster vaccination was higher in our study (89.6%) compared to the global average (31.4%) [19].

This high level of cautiousness, which we found, especially among more severely affected patients, could be an explanation for the fact that we found only a limited number of hospitalized patients. Another explanation for this could be that patients who were prone to a more severe disease course died due to an earlier, more severe, COVID-19 infection: The global COVID-19-related mortality rate was at its highest in the earlier stages of the pandemic [19], well before our study took place. The reasoning of high-risk patients dying in an earlier stage of the pandemic could also be a hypothesis for the remarkable finding that six of the hospitalized patients were female, given that male sex has been identified as a risk factor for a more severe COVID-19 disease course [20, 21]. Overall the results seem to show that patients with Pompe disease suffered a mild COVID-19 infection, although a considerable amount of patients did experience a deterioration of their Pompe disease.

Our study also showed a considerable impact of the COVID-19 pandemic, with interruptions in treatment or care and worsening of patients’ health. In our study population, a cessation of ERT due to the pandemic was more common in patients residing in France. This corresponds with the uncertainty of ERT treatment during the pandemic already having been described in a French population [34]. This study concluded that interruption of ERT, even as short as a few months (mean 2.2 months), worsens a patient’s motor and respiratory function. Patients showed a significant mean deterioration of 37 m in the 6-min walk test and a mean loss of 210 ml of forced vital capacity, without ad integrum restoration after 3 months of ERT restart. A similar (negative) impact of interruption of ERT has been seen in Germany, where the mean time of ERT interruption was 49.9 days [35]. Hence, cessation of ERT is a serious consequence of the pandemic.

Also, 27.7% of patients reported worsening of their disease due to the pandemic. A study conducted in 363 patients with several NMDs, including 8 patients with Pompe disease, has also shown that patients with different NMDs experience disease worsening because of the pandemic, with up to 44% (15/34 patients) of patients with limb–girdle muscular dystrophy reporting this [29]. In the study mentioned, none of the 8 included Pompe patients reported worsening of their disease because of the pandemic. However, this study was conducted during the first 4 months of the pandemic, no COVID-19 infections were reported yet in the patients with Pompe disease and the long-term effects of the pandemic were incalculable at the time. In our study, worsening of overall health with respect to Pompe disease was less frequently reported by mildly affected patients, possibly due to these patients having more residual muscle function compared to more affected patients. This might contribute to mildly affected patients experiencing a smaller impact of deterioration.

Noticeably, an increase in mental complaints (e.g., experiencing possible symptoms of anxiety or depression or experiencing problems with concentrating or sleeping) was reported in our study (55.4%) and this was, surprisingly, more frequently reported by mildly affected patients. An explanation could be that mildly affected patients, because of their substantial residual muscle function, are more likely to be less limited in daily life by Pompe disease. It could be plausible that mildly affected patients were therefore more impacted by the pandemic and its associated control measures. The increase in mental complaints has also been described in the literature regarding the psychosocial impact on other NMDs, such as spinal muscular atrophy, Duchenne muscular dystrophy and congenital muscular dystrophy [30, 31].

Lastly, in our study population, 43.4% of patients had experienced an increased feeling of loneliness and/or social isolation as a result of the pandemic and/or lockdown. A larger proportion of female patients and patients under 65 years of age indicated being more lonely and/or isolated. This is in line with the literature regarding loneliness in the general German population, which concluded higher loneliness scores in 2020 (start of COVID pandemic) compared to 2018, as well as in women and younger groups [38].

This study has some limitations. First, this study is based on a patient-reported questionnaire, meaning medical records to verify the diagnosis were not available. It is, however, unlikely that the patients included in this study are not diagnosed with Pompe disease, since they had to be associated with a national or international patient organization to be recruited and 94.4% of patients indicated they receive ERT, or received ERT in the past, which requires a confirmed diagnosis. Also, because the outcomes are patient-reported, this meant that the deterioration of a patient’s condition could not be objectified. Nonetheless, the goal of this study was to gain insight in the patients’ experience so this is believed to be inessential.

Furthermore, the study population consisted of 144 (42.2%) wheelchair-dependent and 181 (52.9%) ventilator-dependent patients. The proportion of patients that are wheelchair-dependent corresponds to the literature based on the International Pompe Survey, where the number of wheelchair-dependent patients ranged from 34.1 to 45.0% [36, 39]. With regard to ventilator dependency, our population consisted of a larger proportion ventilator-dependent patients than previously described in studies (range 26.0–41.4%) [36, 39]. This could result in a slight overrepresentation of more severely affected patients in this study. Also, more severely affected patients are possibly more likely to be affiliated with a patient organization and to be more motivated to participate in studies. Nevertheless, in this study the spectrum of the patients’ severity of disease varied from no complaints in daily life to wheelchair and ventilator dependency. Hence, we believe we covered the entire spectrum of patients.

Last, it is possible that patients who are more affected by a COVID-19 infection or the pandemic were more likely to respond, which may generate participation bias. This might have resulted in an overestimation of the effects of the COVID-19 infection, the pandemic and its associated control measures on patients with Pompe disease. As mentioned before, it could also be that patients with a more severe COVID-19 infection died. However, the range in symptoms of an infection varied from asymptomatic to the need of respiratory support and the impact of the pandemic also varied from no impact to several mental complaints. Again, we believe this study covered the entire spectrum of patients.

In conclusion, this one-time, cross-sectional survey highlighted that COVID-19 infection(s), the pandemic and its associated control measures resulted in both a—variable but considerable—physical and mental impact on patients with Pompe disease. The results reinforce the need for continued—personalized—care and social engagement, regardless of the severity of the—chronic—disease, during difficult times such as the COVID-19 pandemic and are important to be taken into account in pandemic preparedness protocols to minimize the impact.

### Supplementary Information

Below is the link to the electronic supplementary material.

Supplementary file1 (PDF 818 KB)

## Declarations

### Rights and permissions

Anonymized data are available from the corresponding author on request by a qualified academic investigator. Data transfer will be in agreement with EU legislation on the general data protection regulation and decisions by the Ethical Review Board of the Erasmus  MC University Medical Center.
