# Supplementary material for: The impact of COVID-19 infection, the pandemic and its associated control measures on patients with Pompe disease
Source: J Neurol. 2023 Nov 20;271(1):32–45. doi: 10.1007/s00415-023-11999-2 (PMC10769914; doi:10.1007/s00415-023-11999-2)
Supplement: Supplementary file 1 — Supplementary file1 (PDF 818 KB) [file 415_2023_11999_MOESM1_ESM.pdf]

The impact of COVID-19 infection, the pandemic and its associated control measures on patients with Pompe Disease

Journal of Neurology

Maudy T.M. Theunissen, MD[a], Renee M. van den Elsen[a], Tiffany L. House[b], Brad Crittenden[b], Pieter A. van Doorn, MD, PhD[a], Ans T. van der Ploeg, MD, PhD[c], Michelle E. Kruijshaar, PhD[c], Nadine A.M.E. van der Beek, MD, PhD[a]

[a] Department of Neurology, Center for Lysosomal and Metabolic Diseases, Erasmus MC, University Medical Center Rotterdam, The Netherlands

[b] on behalf of the International Pompe Association

[c] Department of Pediatrics, Center for Lysosomal and Metabolic Diseases, Erasmus MC - Sophia Children's Hospital, University Medical Center Rotterdam, The Netherlands

Corresponding Author: Nadine A.M.E. van der Beek (n.beek@erasmusmc.nl)

One-time COVID-19 questionnaire

for patients 16 years and older

Introduction

This is a one-time questionnaire concerning the effects of COVID-19 (SARS-CoV-2) and the pandemic on the lives of patients with Pompe disease around the world.

This one-time questionnaire consists of 3 parts:

- A: Overall health with respect to Pompe disease (relating to your current complaints regarding Pompe disease)
- B: COVID-19 specific questions (relating to your vaccination status, precautions taken and history of (possible) infection(s) with COVID-19)
- C: Effects of the COVID-19 pandemic (relating to the effects of the pandemic on you and your health care)

Most questions are answered by ticking the one box that best describes your answer (select one). Other questions are answered by ticking all the answer options that apply to you (check all that apply).

The questionnaire should be filled out completely. Please take all the time you need to answer the questions. Filling in this questionnaire will take approximately 20 minutes of your time. The questionnaire ends with a conclusion where we would appreciate any feedback or suggestions.

We thank you in advance for your cooperation; it is much appreciated.

General identifying information

1. Date of completion: |____| |_____| |________|
   DD MM YYYY

2. Month and year of birth: |_____| |________|
   MM YYYY

3. Sex: ☐ Male ☐ Female ☐ Other

4. Country of residence: ___________________________

5. Age of onset symptoms (Pompe disease): |_____|

6. Age of diagnosis (Pompe disease): |_____|

7. Are you currently being treated with enzyme replacement therapy (ERT)? (select one)
   ◯ No, I have never been treated with ERT
   ◯ No, but I have been treated with ERT in the past
   ◯ Yes

8. Are you currently being treated with an experimental therapy?
   ◯ No → continue to section A
   ◯ Yes

   8a. Which experimental therapy do you currently receive?: _______________________________________

A. Overall health with respect to Pompe disease

The following questions concern how much you are currently affected by Pompe disease

1. How would you rate your current overall health with respect to Pompe disease? (select one)

Excellent	*Very good*	*Good*	*Fair*	*Poor*
☐	☐	☐	☐	☐

2. Do you currently use a wheelchair or mobility scooter? (select one)
○ No → continue to question 2b
○ Yes, sometimes
○ Yes, always
2a. Since when have you been using a wheelchair or mobility scooter approximately (part- or full-time)?
|________| *(year)* → continue to question 3
2b. Why do you currently NOT use a wheelchair or mobility scooter? (select one)
○ I do not need a wheelchair or mobility scooter
○ I do not have access to a wheelchair or mobility scooter (e.g. because my health care provider does not fund it), but I do need one according to my clinician
○ I do not want to use a wheelchair or mobility scooter, but I do need one according to my clinician

3. Are you currently able to walk (with or without of the use of the walking aids)? (select one)
○ No → continue to question 4
○ Yes
3a. Do you currently use aids for walking (besides a wheelchair or mobility scooter)? (select one)
○ No
○ Yes → continue to question 3c.
3b. Why do you currently NOT use aids for walking? (select one)
○ I do not need aids for walking → continue to question 4
○ I do not have access to walking aids (e.g. because my health care provider does not fund it), but I do need them according to my clinician → continue to question 4
○ No, I do not want to use walking aids, but I do need them according to my clinician → continue to question 4
3c. Which walking aids do you currently use? (check all that apply) Please also indicate, for the answers that you checked, in which year you approximately started using these aids.
☐ Walking frame / zimmer frame |________| *(year)*
☐ Nordic walking or other poles |________| *(year)*
☐ Other, specify: ________________________ |________| *(year)*

4. Please indicate whether you are currently experiencing, or have experienced in the last month, any of the following breathing problems (check all that apply). Please also indicate, for the answers that you checked, how often this has occurred.

☐ Shortness of breath after heavy exercise	○ Sometimes	○ Often	○ Always
☐ Shortness of breath after a small amount of exercise	○ Sometimes	○ Often	○ Always
☐ Shortness of breath at rest	○ Sometimes	○ Often	○ Always
☐ Shortness of breath when lying down	○ Sometimes	○ Often	○ Always
☐ Pneumonia or airway infection	○ One time	○ Two times	○ More than twice
☐ None			

5. Do you currently use any breathing aids (ventilation)? (select one)
   ○ No → continue to question 5f
   ○ Yes

   5a. Which of the following breathing aids (ventilation) do you use? (check all that apply)
   - ☐ Non-invasive ventilation using a mask (e.g. mouth-nose, full face or nose mask)
   - ☐ Non-invasive ventilation using a mouth piece (ventilation on demand)
   - ☐ Invasive ventilation (endotracheal ventilation) → continue to section B

   5b. Which non-invasive ventilation do you use? (check all that apply)
   - ☐ CPAP
   - ☐ BiPAP/VPAP
   - ☐ Other, specify: ________________________
   - ☐ I don't know

   5c. When do you use these breathing aids? (check all that apply)
   - ☐ During/after exercise
   - ☐ During the daytime
   - ☐ During the night-time (sleeping)

   5d. How many hours per day in total (add up the time during the day and night) do you approximately use these breathing aids?

   Total number of hours: ________ per day

   5e. Since when have you approximately been using aids to help with breathing?

   |________| *(year)*

   ➔ *<u>Continue to section B</u>*

   5f. Why do you currently NOT use any breathing aids? (select one)
   ○ I do not need breathing aids
   ○ I do not have access to breathing aids (e.g. because my health care provider does not fund it), but I do need it according to my clinician
   ○ I do not want to use breathing aids, but I do need it according to my clinician

B. COVID-19 Specific Questions

The following questions concern your vaccination status, precautions taken and history of (possible) infection(s) with COVID-19

<u>Vaccination status</u>

1. Have you been vaccinated against COVID-19? (select one)
   ○ No → continue to question 5
   ○ Yes

2. Did you receive all recommended doses in your primary series of COVID-19 vaccines? (select one)
   ○ No, I only received 1 dose of a 2-dose vaccination
   ○ Yes, I received all recommended doses

3. When did you get your <u>first</u> COVID-19 vaccination?

   |_____| |_______|
   MM YYYY

4. Did you receive a booster dose? (select one)
   ○ No, I did not receive a booster dose
   ○ Yes, I received one booster dose
   ○ Yes, I received two or more booster doses

Precautions

5. What type of precautions did you take at the **beginning** of the COVID-19 pandemic (spring 2020)? (check all that apply)
   ☐ Full quarantine (only essential contact)
   ☐ Mask when out in public or around those not in household
   ☐ Keep 1.5 meter distance
   ☐ Only meet outdoors
   ☐ No precautions
   ☐ Other, specify: ________________________

6. What type of precautions do you **currently** take against COVID-19? (check all that apply)
   ☐ Full quarantine (only essential contact)
   ☐ Mask when out in public or around those not in household
   ☐ Keep 1.5 meter distance
   ☐ Only meet outdoors
   ☐ No precautions
   ☐ Other, specify: ________________________

7. Were you able to work from home during the COVID-19 pandemic? (select one)
   ◯ I do not have a job/volunteer
   ◯ No, I was an essential healthcare worker and allowed to work elsewhere still
   ◯ No, I was an essential non-healthcare worker and allowed to work elsewhere still
   ◯ Yes

8. Besides yourself, how many other people do you live with in your house-hold?

   |____| (fill in a number)

Infection with COVID-19

9. Have you had an infection with COVID-19? (select one)
   ◯ No → continue to section C
   ◯ Yes
   ◯ I think I had COVID-19, but I did not get tested → continue to section C
   ◯ I don't know → continue to section C

In the case of having had multiple COVID-19 infections: questions 10 to 17 concern your *first* infection with COVID-19.

10. When did you first have a positive test?

    |_____| |________|
    MM YYYY

11. What were your primary symptoms? (check all that apply)
    ☐ Nose cold (e.g. runny nose, sneezing)
    ☐ Significant and persistent coughing
    ☐ Fever or increased temperature
    ☐ Sore throat
    ☐ Difficulty breathing
    ☐ Sudden loss of smell and/or taste
    ☐ Headache
    ☐ Tiredness
    ☐ Muscle pain or other pain complaints
    ☐ Other, specify: _____________________________
    ☐ I didn't have any complaints

12. Did you get admitted to the hospital because of this COVID-19 infection? (select one)
   ○ No → continue to question 13
   ○ Yes
      12a. Did you receive (additional) respiratory support in the hospital? (select one)
         ○ No
         ○ Yes
      12b. Were you admitted to the intensive care unit (ICU)? (select one)
         ○ No
         ○ Yes

13. Did you need to rehabilitate in a rehabilitation center or nursing home (or similar institution) after having a COVID-19 infection? (select one)
   ○ No
   ○ Yes

14. How long did it take you to recover from the COVID-19 infection? (select one)
   ○ < 4 weeks → continue to question 15
   ○ 4-8 weeks
   ○ 8-12 weeks
   ○ > 12 weeks
   ○ I still have not fully recovered
      14a. Which residual complaints do or did you experience? (check all that apply)
         ☐ Tiredness
         ☐ Coughing
         ☐ Shortage of breath
         ☐ Lost or changed sense of smell and/or taste
         ☐ Headache
         ☐ Muscle pain or other pain complaints
         ☐ Palpitations
         ☐ Mental health issues (e.g. anxiety or depression)
         ☐ Other, specify: ______________________________

15. What was the effect of the COVID-19 infection on your overall condition, compared to before this infection? (select one)
   ○ The infection had no effect on my condition
   ○ My condition has been made somewhat worse
   ○ My condition has been made much worse
   ○ Other, specify: ______________________________

16. What was the effect of the COVID-19 infection on your respiratory status, compared to before this infection? (select one)
   ○ The infection had no effect on my respiratory status
   ○ My respiratory status has been somewhat worse
   ○ My respiratory status has been made much worse
   ○ Other, specify: ______________________________
      16a. Was there a need to adjust the settings of your breathing aid(s) (ventilation) or did you need to use it more hours per day? (select one)
         ○ No, I don't use breathing aids → continue to question 17
         ○ No, there were no changes necessary → continue to question 17
         ○ Yes, I started using breathing aids due to the COVID-19 infection
         ○ Yes, the settings of the breathing aid(s) changed
         ○ Yes, I did need the breathing aid(s) more hours per day
         ○ Yes, the settings of the breathing aid(s) changed AND I did need the breathing aid(s) more hours per day
      16b. Were these changes in ventilation temporary or permanent? (select one)
         ○ Temporarily
         ○ Permanently

17. What was the effect of the COVID-19 infection on your mobility status, compared to before this infection? (select one)
    ○ The infection had no effect on my mobility status
    ○ My mobility status has been made somewhat worse
    ○ My mobility status has been made much worse
    ○ Other, specify: ______________________________

18. Did you have COVID-19 more than once? (select one)
    ○ No, I only got COVID-19 once → continue to section C
    ○ Yes, I got COVID-19 two or more times

<u>The following questions (19 to 26) concern your *second* infection with COVID-19.</u>

19. When did you first have a positive test?

    |_____| |________|
    MM YYYY

20. What were your primary symptoms? (check all that apply)
    ☐ Nose cold (e.g. runny nose, sneezing)
    ☐ Significant and persistent coughing
    ☐ Fever or increased temperature
    ☐ Sore throat
    ☐ Difficulty breathing
    ☐ Sudden loss of smell and/or taste
    ☐ Headache
    ☐ Tiredness
    ☐ Muscle pain or other pain complaints
    ☐ Other, specify: ______________________________
    ☐ I didn't have any complaints

21. Did you get admitted to the hospital because of this COVID-19 infection? (select one)
    ○ No → continue to question 22
    ○ Yes
        21a. Did you receive (additional) respiratory support in the hospital? (select one)
            ○ No
            ○ Yes
        21b. Were you admitted to the intensive care unit (ICU)? (select one)
            ○ No
            ○ Yes

22. Did you need to rehabilitate in a rehabilitation center or nursing home (or similar institution) after having a COVID-19 infection? (select one)
    ○ No
    ○ Yes

23. How long did it take you to recover from the COVID-19 infection? (select one)
    ○ < 2 weeks → continue to question 24
    ○ 2-4 weeks → continue to question 24
    ○ 4-8 weeks
    ○ 8-12 weeks
    ○ > 12 weeks
    ○ I still have not fully recovered

23a. Which residual complaints do or did you experience? (check all that apply)
- ☐ Tiredness
- ☐ Coughing
- ☐ Shortage of breath
- ☐ Lost or changed sense of smell and/or taste
- ☐ Headache
- ☐ Muscle pain or other pain complaints
- ☐ Palpitations
- ☐ Mental health issues (e.g. anxiety or depression)
- ☐ Other, specify: ______________________________

24. What was the effect of the COVID-19 infection on your overall condition, compared to before this infection? (select one)
- ○ The infection had no effect on my condition
- ○ My condition has been made somewhat worse
- ○ My condition has been made much worse
- ○ Other, specify: ______________________________

25. What was the effect of the COVID-19 infection on your respiratory status, compared to before this infection? (select one)
- ○ The infection had no effect on my respiratory status
- ○ My respiratory status has been somewhat worse
- ○ My respiratory status has been made much worse
- ○ Other, specify: ______________________________

25a. Was there a need to adjust the settings of your breathing aid(s) (ventilation) or did you need to use it more hours per day? (select one)
- ○ No, I don't use breathing aids → continue to question 26
- ○ No, there were no changes necessary → continue to question 26
- ○ Yes, I started using breathing aids due to the COVID-19 infection
- ○ Yes, the settings of the breathing aid(s) changed
- ○ Yes, I did need the breathing aid(s) more hours per day
- ○ Yes, the settings of the breathing aid(s) changed AND I did need the breathing aid(s) more hours per day

25b. Were these changes in ventilation temporarily or permanently? (select one)
- ○ Temporarily
- ○ Permanently

26. What was the effect of the COVID-19 infection on your mobility status, compared to before this infection? (select one)
- ○ The infection had no effect on my mobility status
- ○ My mobility status has been made somewhat worse
- ○ My mobility status has been made much worse
- ○ Other, specify: ______________________________

27. Did you have a COVID-19 infection more than twice? (select one)
- ○ No, I only got COVID-19 two times → continue to section C
- ○ Yes, I got COVID-19 three or more times

28. Were the symptoms from your later COVID-19 infections different than the symptoms you experienced during your first and second infection? (select one) If *yes*, please describe which new symptoms you experienced and whether in general the symptoms were more or less severe than before.
- ○ No, I had similar symptoms
- ○ Yes, please describe: ____

____

C. Effects of the COVID-19 pandemic

The following questions concern the effects of the COVID-19 pandemic in general and its measures on you and your health care

1. Compared to the start of the pandemic (spring 2020), how is your overall health with respect to Pompe disease now? (select one)
   ○ Much better
   ○ Somewhat better
   ○ About the same
   ○ Somewhat worse
   ○ Much worse

2. Do you think the pandemic and/or lockdown has changed how much you are affected by Pompe disease? (select one)
   ○ No
   ○ Yes, somewhat
   ○ Yes, a lot
   ○ Other, specify: ______________________________

3. Do you feel your level of physical activity has changed as a result of the pandemic and/or lockdown? (select one)
   ○ No, I sustained the same level of activity
   ○ Yes, somewhat
   ○ Yes, a lot
   3a. Do you feel this directly influenced your mobility? (select one)
   ○ No
   ○ Yes

4. How did your lifestyle change in the last two years as a result of the pandemic? For each subject please tick the box that best describes your answer.
   - Exercise ☐ More ☐ Same ☐ Less ☐ I don't know
   - Alcohol use ☐ More ☐ Same ☐ Less ☐ I don't drink alcohol ☐ I don't know
   - Smoking ☐ More ☐ Same ☐ Less ☐ I don't smoke ☐ I don't know
   - Hours of sleep ☐ More ☐ Same ☐ Less ☐ I don't know
   - Intake of fruit ☐ More ☐ Same ☐ Less ☐ I don't know
   - Intake of vegetables ☐ More ☐ Same ☐ Less ☐ I don't know
   - Snacks (cookies, crisps, cakes etc.) ☐ More ☐ Same ☐ Less ☐ I don't know
   - Being aware of food habits ☐ More ☐ Same ☐ Less ☐ I don't know
   - Other lifestyle changes that I would like to share: ____
   ____

5. Did you experience any interruptions or delays in *disease specific treatment* (e.g. in enzyme replacement therapy (ERT)) as a result of the pandemic? (select one)
   ○ No → continue to question 9
   ○ Yes, my ERT infusions were interrupted (not in a clinical trial) → continue to question 6
   ○ Yes, my ERT infusions were interrupted (in a clinical trial) → continue to question 6
   ○ Yes, the start of my ERT therapy was delayed → continue to question 7
   ○ Yes, treatment that was not ERT was delayed or interrupted → continue to question 8

6. How many infusions were missed in total?

   |____| (fill in a number) → If you don't know the exact number please give us a rough estimate.
   6a. Were these missed infusions consecutive or at different times? (select one)
   ○ Consecutive
   ○ At different times

7. Do you feel this delay or interruption has negatively affected your condition? (select one)
   - ◯ No → continue to question 9
   - ◯ Yes, please explain: ____
   ____→ continue to question 9

8. Which treatment was delayed or interrupted and how? Please explain: ______________________________
   ____
   ____
   8a. Do you feel this delay or interruption has negatively affected your condition? (select one)
   - ◯ No
   - ◯ Yes, please explain: ____
   ____

9. Did you experience a change in medical appointments or examinations as a result of the pandemic? (select one)
   - ◯ No → continue to question 10
   - ◯ Yes

   9a. Which changes in medical appointments or examinations did you experience as a result of the pandemic? (check all that apply)
   - ☐ Reduced frequency
   - ☐ Medical consultations by telephone or web
   - ☐ Other, specify: ____________________________________

10. Did you experience a change in the amount of physical therapy as a result of the pandemic? (select one)
    - ◯ No, I maintained the same level of physical therapy → continue to question 11
    - ◯ Yes, my physical therapy paused temporarily once
    - ◯ Yes, my physical therapy paused temporarily more than once
    - ◯ Other, specify: ______________________________ → continue to question 11

    10a. How long was your physical therapy paused for? Please specify: ________________________
    ____

11. Do you currently have care-giver help? (select one)
    - ◯ No → continue to question 12
    - ◯ Yes

    11a. When did you start having care-giver help? (select one)
    - ◯ Before the pandemic
    - ◯ During the pandemic and it started when I needed it
    - ◯ During the pandemic and it started later than I needed because of the pandemic
    - ◯ During the pandemic and it started later than I needed because of other reasons than the pandemic

    11b. Did you have reduced visits as a result of the pandemic? (select one)
    - ◯ No → continue to question 12
    - ◯ Yes

    11c. What was the effect of having reduced visits? (check all that apply)
    - ☐ I experienced increased loneliness and/or isolation
    - ☐ I was less active
    - ☐ I got less help with activities of daily living (e.g. getting dressed or showering
    - ☐ I got less medical care (e.g. keeping track of medicine)
    - ☐ I got less help with grocery shopping and/or cooking
    - ☐ My housework was behind
    - ☐ I got less help with managing personal services (e.g. talking to doctors or paying bills)
    - ☐ Other, specify: _________________________

12. What was the effect of the COVID-19 pandemic and/or lockdown on your mental health? (select one)
   ○ It hasn't affected me at all → continue to question 13
   ○ I am somewhat affected
   ○ I am much affected
   ○ It fluctuates depending on my mood

   12a. How did the COVID-19 pandemic and/or lockdown affect your mental health? (check all that apply). Please indicate how you felt during the pandemic compared to before the pandemic.
   - ☐ I felt more nervous, restless or tense
   - ☐ I felt more sad, tearful, empty or hopeless
   - ☐ I had an increased sense of impeding danger, panic or doom
   - ☐ I had more trouble concentrating and thinking
   - ☐ I had more trouble sleeping
   - ☐ I had more difficulty controlling worry
   - ☐ I had more angry outbursts, irritability or frustration
   - ☐ I had reduced appetite or increased cravings for food
   - ☐ Other, specify: ______________________________

13. What have you done to cope with the pandemic and/or lockdown? (check all that apply)
   - ☐ I talk to friends and family
   - ☐ I do meditation and/or yoga
   - ☐ I go to online therapy sessions
   - ☐ I visited a social worker and/or psychologist
   - ☐ I perform religious practices
   - ☐ I pursue my hobbies
   - ☐ Other, specify: ______________________________

14. Have you experienced increased loneliness and/or isolation as a result of the pandemic and/or quarantine? (select one)
   ○ No → continue to the conclusion
   ○ Yes

   14a. What have you done to cope with increased loneliness and/or isolation? (check all that apply)
   - ☐ Increased Social Media use (e.g. Facebook, Instagram, Twitter, TikTok etc.)
   - ☐ Increased amount of time spent calling friends and family
   - ☐ Increased use of videocalls (e.g. Zoom, WhatsApp, FaceTime, Teams etc.)
   - ☐ Increased amount of time spent messaging friends and family
   - ☐ Increased time spent on entertainment (reading, watching TV etc.)
   - ☐ Started a new hobby or revisited an old hobby
   - ☐ I go to online therapy sessions
   - ☐ I perform religious practices
   - ☐ Other, specify: ______________________________

Conclusion

Is there anything you would like to share that you do not feel was already covered?

____

____

____

____

____

____
